# Editorial: Judgment and Decision Making Under Uncertainty: Descriptive, Normative, and Prescriptive Perspectives

**DOI:** 10.3389/fpsyg.2019.01506

**Published:** 2019-07-02

**Authors:** David R. Mandel, Gorka Navarrete, Nathan Dieckmann, Jonathan Nelson

**Affiliations:** ^1^Intelligence, Influence and Collaboration Section, Defence Research and Development Canada, Toronto, ON, Canada; ^2^Department of Psychology, York University, Toronto, ON, Canada; ^3^Center for Social and Cognitive Neuroscience, School of Psychology, Universidad Adolfo Ibáñez, Santiago, Chile; ^4^School of Nursing, Oregon Health & Science University, Portland, OR, United States; ^5^Decision Research, Eugene, OR, United States; ^6^School of Psychology, University of Surrey, Guildford, United Kingdom; ^7^iSearch Group, Max Planck Institute for Human Development, Berlin, Germany

**Keywords:** judgment, decision-making, uncertainty, cognition, psychology

*Judgment and Decision Making Under Uncertainty: Descriptive, Normative, and Prescriptive Perspectives* was motivated by our interest in better understanding why people judge and decide as they do (descriptive perspective), how they ideally ought to judge and decide (normative perspective), and how their judgment and decision-making processes might be improved in practice (prescriptive perspective). We sought papers that addressed some aspect of judgment and decision making from one or more of these three theoretical perspectives. We further sought contributions that examined judgment and decision making under conditions of uncertainty, which we intentionally left loosely defined. Our focus on uncertainty reflects the fact that the vast majority of decisions people make in life are not made under conditions of complete certainty, and the uncertainties may be more or less well-defined. Indeed, different components of a single judgment or decision may have multiple uncertainties associated with it, some of which may be fuzzier than others. Following our call for papers, we received 32 submissions, 17 of which were accepted. The latter set comprises this book. There are 11 original research articles, 2 hypothesis and theory articles, 2 perspectives, and 1 book review and systematic review each.

This book, the culmination of a *Frontiers in Psychology* Cognition section Research Topic, is closely related to an earlier Research Topic and book entitled *Improving Bayesian Reasoning: What Works and Why* that two of us edited (Navarrete and Mandel, 2016). The current book shows strong continuity with its conceptual cousin. Several papers address aspects of Bayesian judgment or reasoning. In “Why can only 24% solve Bayesian reasoning problems in natural frequencies: Frequency phobia in spite of probability blindness,” Weber et al. find that, despite the benefit to accuracy conferred by representing statistical information in natural frequencies, many participants translate natural frequencies back into probabilities. This appears to be an important factor in explaining the low rates of accurate judgment. In “How to improve performance in Bayesian inference tasks: A comparison of five visualizations,” Böcherer-Linder and Eichler investigate the effectiveness of three graphical properties of visualizations: area-proportionality, use of discrete and countable statistical entities, and graphical transparency of the nested-sets structure. They find that the primary factor contributing to performance in Bayesian reasoning problems was graphically representing the nested-set structure of the problem in a transparent manner, followed secondarily by representing discrete objects. In “What eye-tracking can tell us on statistical reasoning—An empirical study on tree diagrams and 2 × 2 tables,” Bruckmaier et al. use eye tracking to shed light on the reasons for errors in probabilistic judgment. They show that different reasoning processes can account for errors that look similar behaviorally. Conversely, errors that look different may stem from common reasoning processes. In “Bayesian revision vs. information distortion,” Russo explains how a normative requirement of Bayesian reasoning—namely, that likelihoods should be independent of the prior probability—is routinely violated in all but the most contrived judgment problems where such violations are designed to be impossible. The violations, Russo argues, occur because people strive for coherence and therefore seek to bring new evidence in line with their prior beliefs. Evidently, the pursuit of coherence can at times signal its downfall. Finally, in “Metacognitive myopia in hidden-profile tasks: the failure to control for repetition biases,”  Fiedler et al. address an issue that is conceptually related to updating processes when confronting correlated evidence. They find that mere repetition of information over time (which can be thought of as a form of correlated evidence) can undermine the optimal use of information that is distributed across members of a collective. As they aptly point out, given the vast opportunities for information repetition to trigger such biases, it is vital that metacognitive monitoring takes place, and yet their results indicate that people have a difficult time doing so.

A second set of papers tackles uncertainty from several fresh vantage points. In “The psychology of uncertainty and three-valued tables,” Baratgin et al. examine people's three-valued (i.e., certainly true, certainly false, or neither) truth tables for several natural language connectives. Comparing multiple three-valued logics, they find that de Finetti's (1936/1995) three-valued system provides the best descriptive model. Their work on the de Finettian “Level 1,” in which uncertainty is distinguished from certain states, represents a long neglected bridge between Level 0 (binary logic) and Level 2 (studies of probability judgment in which uncertainty is quantified). In “Imprecise uncertain reasoning: a distributional approach,” Kleiter develops an approach to using mental probability logic in concert with beta distributions, copulas, vines, and stochastic simulation to model imprecise and uncertain reasoning. A key finding from his analysis of several classic judgment problems is that the probabilities inferred from different logical inference forms can be so close as to make their distinction impossible in psychological research, a result that has striking implications for the interpretation of evidence in judgment research. In “The role of type and source of uncertainty on the processing of climate models projections,” Benjamin and Budescu examine how people's interpretations of climate change forecasts from multiple experts are influenced by two sources of uncertainty: imprecision (i.e., the width of the confidence interval around a single estimate) and conflict (the extent to which experts disagree). They find that participants were more averse to conflict and reacted more positively to communications that reflect imprecision. Their results show that people's perceptions of competing climate change forecasts are affected by a complex interaction between sources of uncertainty and task characteristics. This set of papers is nicely rounded out by Mousavi's book review of Machina and Viscusi's *Handbook of the Economics of Risk and Uncertainty*.

A third set of papers addresses topics in decision-making under uncertainty. In “Meta-analytic evidence for a reversal learning effect on the Iowa Gambling Task in older adults,” Pasion et al. report a systematic review of studies examining older-adult decision-making on the Iowa Gambling Task. They find evidence of a significant reversal learning effect across blocks of the task, which suggests that older adults show adaptive decision making as they gain experience with the outcomes. In “Cognitive style and frame susceptibility in decision making,” Mandel and Kapler examine the predictive effect of several cognitive style and performance measures on frame susceptibility or “going with the frame.” They do not find such factors to be predictive of frame susceptibility and they question the theoretical claim that individuals who are prone to a less deliberate, or more intuitive, thinking style are more susceptible to framing effects. In “Too worried to judge: On the role of perceived severity in medical decision-making,” Colomé et al. examine content effects on recommendations for medical treatments. They find that worry affects recommendations only in the higher severity context (cancer), whereas consideration of disease likelihood given a positive test result played a greater role in the lower severity context (hypertension). In “The reciprocal relationships between escalation, anger, and confidence in investment decisions over time,” Jackson et al. show in an escalation of commitment task, where money had to be invested in different rounds in a never-ending project, people tend to escalate through all rounds. However, as they do, their confidence decreases and anger increases, thus shedding light on the experiential side of this well-documented phenomenon. In “Does fear increase search effort in more numerate people? An experimental study investigating information acquisition in a decision from experience task,” Traczyk et al. examine the role of numeracy and emotion of fear on search policy and choice in a decision from experience task. Both numeracy and fear were related to increased information sampling, although the effect of fear was restricted to a more numerate subsample. Their results shed light on the interaction between numeracy and integral emotion in decisions from experience.

Last but not least, three papers draw on decision science to shed light on professional practices in forensics and national security intelligence. In “Decisional dimensions in expert witness testimony—a structural analysis,” Biedermann and Kotsoglou integrate decision theory with current practices in forensic science for the use of expert witness testimony. The authors review current theoretical understanding of the expert witness testimony process and then discuss a decision-theoretic framework including real-world examples. In “Better together: reliable application of the post-9/11 and post-Iraq US intelligence tradecraft standards requires collective analysis,” Marcoci et al. turn their attention to the US intelligence community's analytic tradecraft standards by asking whether raters can interpret the standards reliably as they pertain to intelligence products. Overall, the reliability of single raters was poor. Having important prescriptive implications for quality control within the intelligence community, Marcoci et al. find that a group of three or more raters is needed to provide reliable assessments of the quality of intelligence products. Finally, in “Correcting judgment correctives in national security intelligence,” Mandel and Tetlock argue that the intelligence community's prescriptions for improving analysts' intelligence assessments—namely, their judgments under uncertainty—could be substantially improved by scientifically testing the effectiveness of proposed methods; something rarely done. Drawing on decision science, Mandel and Tetlock argue that current methods might not only fail to improve analysts' judgments, they may in fact be making intelligence assessments less reliable, coherent and accurate.

We hope the reader will find this book informative, thought provoking, and of practical and theoretical value.

## Author Contributions

DM wrote the editorial. GN, ND, and JN provided input and constructive feedback.

### Conflict of Interest Statement

The authors declare that the research was conducted in the absence of any commercial or financial relationships that could be construed as a potential conflict of interest.
